# Effect of Serving Plate Types and Color Cues on Liking and Purchase Intent of Cheese-Flavored Tortilla Chips

**DOI:** 10.3390/foods10040886

**Published:** 2021-04-17

**Authors:** Cristhiam E. Gurdian, Damir D. Torrico, Bin Li, Witoon Prinyawiwatkul

**Affiliations:** 1 School of Nutrition and Food Sciences, Louisiana State University Agricultural Center, Baton Rouge, LA 70803, USA; cgurdi3@lsu.edu; 2 Department of Wine, Food and Molecular Biosciences, Faculty of Agriculture and Life Sciences, Lincoln University, Lincoln 7647, New Zealand; damir.torrico@lincoln.ac.nz; 3 Department of Experimental Statistics, Louisiana State University Agricultural Center, Baton Rouge, LA 70803, USA; bli@lsu.edu

**Keywords:** serving plate, color cue, liking, purchase intent, tortilla chips

## Abstract

Foods’ overall liking (OL) and purchase intent (PI) are influenced by visual inputs, such as color cues and serving plate types. Cheese-flavored tortilla chips (CFTC) from two formulations (A and B) with a noticeable color difference (∆E = 4.81) were placed on different serving plates (plastic, foam, and paper) and presented monadically to N = 83 consumers using a randomized/balanced block design in two sessions. Consumers evaluated likings of overall visual quality, color, crunchiness, saltiness, overall flavor (OF), and OL using a 9-point-hedonic scale, attribute appropriateness on a 3-point-just-about-right (JAR) scale, and PI using a binomial (Yes/No) scale. Color differences between A and B influenced crunchiness and saltiness liking and perception, which together with OF liking and formulation, mainly determined OL of CFTC. Although having similar fracturability (N) and sodium content, formulation A had higher crunchiness and saltiness likings. PI was influenced by crunchiness, saltiness, and OF liking with 37, 49, and 60% increases in PI odds per liking-unit increase, respectively. Plate type had minimal effect on the sensory liking of CFTC. The brighter and less-yellow color of CFTC could positively influence liking of crunchiness and saltiness, which significantly contributed to OL and PI. These findings are useful to understand consumers’ acceptability and perception of foods when varying visual inputs.

## 1. Introduction

Consumers are influenced by intrinsic and extrinsic cues when evaluating product quality. Intrinsic cues refer to those attributes that are part of the product’s objective nature (e.g., color, aroma, flavor) whereas extrinsic cues (e.g., packaging material, nutritional label, claims) are characteristics that can be altered in the product without changing the objective nature of the product [1].

Product cues can alter expectations, perceptions, emotions, consumption patterns, purchase intent (PI), and other food-related behaviors in consumers. In a previous study, Buhrau and Ozturk [2] found that hedonic expectations and consumption willingness of meals were affected by the format of presentation (text vs. picture) for consumers with low-health consciousness. On the other hand, the health-related perceptions of these consumers remained constant. Improved hedonic perceptions and consumption willingness among consumers with low-health consciousness occurred when meals were presented using the picture format. Bolhuis and Keast [3] investigated the effect of cutlery type (forks vs. spoons) on food intake, reporting that body weight status and cutlery type affected the eating rate of consumers. Fork users tended to consume slower and in lesser amounts than spoon users, who presented a higher body mass index.

Other researchers evaluated the effect of altering the weight, size, color, and shape of cutlery on individuals’ perceptions of sweetness, saltiness, density, value, and overall liking of foods [4]. Lighter spoons yielded higher yogurt density values and liking scores than heavier spoons. Both spoon size and weight (interaction) influenced the perceived sweetness of yogurts. The spoon color influenced yogurt’s taste (increased saltiness scores for the pink yogurt in blue spoon vs. white yogurt in the same spoon), which was also affected by the color of the yogurt. Cutlery shape also altered the perception of cheeses with increased saltiness ratings for those tasted from a knife vs. spoon, fork, or toothpick. Moreover, sensation transference, disconfirmation of expectation, and mood/emotion prompts were suggested as possible underlying mechanisms in modeling an individual’s sensory perception of foods [4].

Color, an important component of foods, brand names, packages, and logos, can be used to convey information, expectations, and overall acceptability of products in consumers’ minds. Such mental scenarios are affected by previous experiences, sociodemographic patterns, and physiological and psychological aspects that govern consumers’ mindsets [5]. Chonpracha, et al. [6] found that increasing the viscosity and yellow/brown color intensity in syrups increased sweetness expectations and reduced syrups’ consumption amounts, without affecting the sensory liking of brewed coffee.

Previous studies showed that extrinsic and intrinsic cues of food stimuli interact dynamically with the subjects’ expectations, perceptions, liking, and PI of products [2,3,7,8,9,10,11]. Zellner, et al. [12] found that the expectations driven by extrinsic color cues varied depending on the product and extrinsic color cues had a lower effect than intrinsic color cues on flavor perceptions.

Literature findings of specific intrinsic or extrinsic cues vary depending on the food stimuli. There are very few studies regarding the effects of extrinsic cues on the acceptability of popular snacks, such as chips and their serving format. Corn chips represent an important market share in the savory snack market valued at over USD 35 billion. In the US more than one in three Americans (including children and adults) consume a savory snack portion per day. This consumption pattern is independent of income level in adults and irrespective of race/ethnic backgrounds and income in children [13]. Most of the published literature discussed the effects of visual cues on improving salty or sweet taste perception. Still, the effect of visual cues on crunchiness perception, a major driver of liking, has not been fully studied yet. Therefore, the research objective of the present study was to understand the effects of serving plate types and products’ colors on the sensory liking, perception, and PI of cheese-flavored tortilla chips (CFTC). Instrumental color and fracturability measurements were conducted on two CFTC formulations from commercially available brands followed by a consumer study evaluating their acceptability and PI when presenting samples from both formulations on each type of serving plate (plastic, foam, and paper).

## 2. Materials and Methods

### 2.1. Materials

Cheese-flavored tortilla chips (CFTC) with formulation A (corn, vegetable oil (corn, canola, and/or sunflower oil), maltodextrin (made from corn), salt, cheddar cheese (milk, cheese cultures, salt, enzymes), whey, monosodium glutamate, buttermilk, romano cheese (part-skim cow’s milk, cheese cultures, salt, enzymes), whey protein concentrate, onion powder, corn flour, natural and artificial flavor, dextrose, tomato powder, lactose, spices, artificial color (yellow 6, yellow 5 and red 40), lactic acid, citric acid, sugar, garlic powder, skim milk, red and green bell pepper powder, disodium inosinate and disodium guanylate) and 7-in-diameter plastic (Chinet, Cut Crystal, Huhtamaki, De Soto, KS, USA) and foam plates (Great Value, Soak-Proof, Walmart Inc., Bentonville, AR, USA) were purchased locally at Walmart Supercenter (Baton Rouge, LA, USA). CFTC with formulation B (whole corn, vegetable oil (contains one or more of the following: cottonseed, corn, canola, soybean and/or sunflower), maltodextrin, salt, dextrose, monosodium glutamate, rice flour, onion powder, cheddar cheese (milk, salt, cultures and enzymes, and disodium phosphate), spices, tomato powder, natural and artificial flavors, yellow cornmeal, artificial colors (red 40, blue 1, yellow 5, yellow 6 lake, yellow 5 lake, red 40 lake), lactic acid, citric acid, garlic powder, sodium diacetate, disodium inosinate and disodium guanylate) and 7-in-diameter paper plates (Party, Greenbrier International, Inc., Chesapeake, VA, USA) were purchased at a Dollar Tree Store (Baton Rouge, LA, USA). Both CFTC (A and B) had a “guaranteed fresh” date until 11–22 January 2018.

### 2.2. Physico-Chemical Analysis

Intact (whole) and uniform (in terms of size and shape) CFTC from both formulations (A and B) were used for the instrumental color and texture (fracturability) characterization. Triplicate samples of CFTC were macerated for 4 min in a Lab Blender 400 model STO 400 (Tekmar Co., Cincinnati, OH, USA) using Whirl-Pack sampling bags (Nasco Co., Fort Atkinson, WI, USA) and analyzed for instrumental color measurement using the petri dish measurement full set CM-A205 in a spectrophotometer model CM-5 (Konica Minolta Inc., Osaka, Japan) in a room illuminated with the same natural light that was used for the consumer tests. An internal white calibration plate was used to standardize the instrument. The resulting L* (0—darkness, 100—lightness), a* (− greenness, + redness), and b* (− blueness, + yellowness) values were subsequently used to calculate the magnitude of total color difference (∆E) [14] between formulations according to Equation (1).

(1) $$\Delta E=\sqrt{{(\Delta L*)}^{2}+(\Delta {a*)}^{2}+(\Delta {b*)}^{2}}$$

where ΔL* = L*_formulation(A)_ − L*_formulation(B)_; Δa* = a*_formulation(A)_ − a*_formulation(B)_; Δb* = b*_formulation(A)_ − b*_formulation(B)_.

Six samples of intact (whole) CFTC from each formulation (A and B) were analyzed for instrumental fracturability (N) using a cylindrical probe with a rounded tip (TA-8, Dia-1/4” or 6.35 mm stainless steel ball) and a crisp fracture support rig located on the heavy-duty platform of a Texture Analyzer (TA.XT.Plus, Texture Technologies Corp., Scarsdale, NY, USA) connected to a 5 Kg load cell. Settings for this compression test were: 1 mm/s, 1 mm/s, and 10 mm/s pre-test, test, and post-test speeds, respectively, 7 mm distance target mode, 5 g trigger force, auto tare mode, and 500 pps data acquisition rate. Fracturability encompasses crunchiness, crispiness, crumbliness, and brittleness. Previous studies have reported a strong positive correlation between instrumental fracturability and sensory crunchiness [15] and fracture testing is among the most suitable techniques for simulating eating [16].

### 2.3. Preparation of Cheese-Flavored Tortilla Chips (CFTC) Samples for Consumer Tests

**Figure 1:**
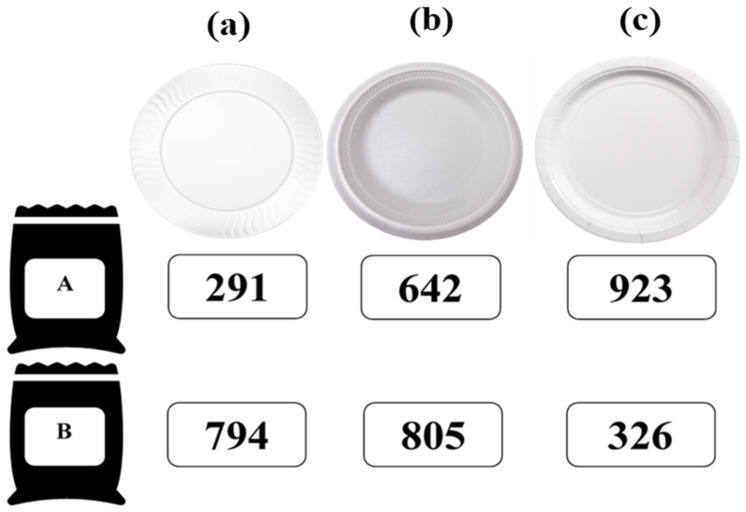
Treatments (cheese-flavored-tortilla chip formulations A and B presented in (**a**) plastic, (**b**) foam, and (**c**) paper plates) and random-three-digit codes used for the consumer tests.

Only intact (whole) CFTC were used in the consumer test. Samples (three chips from each formulation) were placed on each of the three serving plates (constituting the treatments) the same day of the study for the 2-day sessions using CFTC from unopened bags so that samples were evaluated fresh (Figure 1).

### 2.4. Sensory Evaluation

#### 2.4.1. Subjects

A total of N = 83 untrained subjects (42 males and 41 females between 18–65 years old) were recruited from a pool of staff and students at the Louisiana State University (LSU) campus, Baton Rouge, Louisiana on 3 and 6 November 2017. Before their enrollment as panelists, all subjects were screened according to the following criteria: (1) willingness to participate, (2) self-report on no allergies or adverse reactions to the test samples, (3) not having impaired vision/color blindness or taste/smell conditions that would compromise their sensory evaluations, and (4) being regular consumers (at least once per month) of cheese-flavored tortilla chips (CFTC) based on self-reported responses. To participate in the study, subjects agreed with and signed a consent form included in the research protocol approved (IRB # HE 15−9) by the LSU Agricultural Center Institutional Review Board. All participants were also informed of any allergens that may be present in the study: milk/dairy products (from CFTC samples) and gluten (from unsalted crackers used to cleanse the palate). Consumer evaluations took place in partitioned booths equipped with white lights in the Sensory Laboratory at LSU under a controlled environment and a set temperature of 25 °C. Consumers who participated in the sensory evaluation were compensated with a refreshment.

#### 2.4.2. Sensory Procedure

Each panelist evaluated all the treatments (Figure 1) by performing two consumer tests with 3 out of the 6 samples per session. On each session, water and unsalted crackers were provided for panelists before the first sample and in between samples to cleanse their palate. After panelists consented to participate in the tests they were instructed to (1) rate their likings with a 9-point-hedonic scale (left-anchored dislike extremely and right-anchored like extremely) for overall visual quality, color, crunchiness, saltiness, overall flavor (OF), and overall liking (OL), (2) rate their attribute appropriateness perception with a 3-point just-about-right (JAR) scale (left-anchored not enough, mid-anchored JAR and right-anchored too much) for orange color, crunchiness, saltiness, and cheese flavor (CF), and (3) indicate their purchase intent (PI) if the product was commercially available with a binomial scale (Yes or No). Samples’ assignment and their monadic presentation order were balanced and randomized within each session. Random and unique three-digit codes were assigned to each sample regardless of formulation or plate type to avoid influence across samples. All data were collected with Compusense sensory software (Compusense release 5.6, Compusense Inc., Guelph, ON, Canada).

### 2.5. Experimental Design and Statistical Analysis

Two-sample T-tests (*p* ≤ 0.05) were used to compare formulations on instrumental color measurements (L*, a*, b*) and fracturability (N). A Randomized Block Design model with a factorial treatment arrangement (plate type and formulation factors with two-way interactions) was used to investigate the effect of plate type and formulation on the sensory liking of the CFTC using panelists as blocks. Two-way Analysis of Variance (ANOVA) with a mixed-effects (plate type and formulation factors with two-way interactions as fixed effects and panelists as random effects) model and a post-hoc Tukey’s honestly significantly different (HSD) test (*p* ≤ 0.05) were used to assess significant differences in the hedonic ratings of the CFTC. Two-sided Cochran’s Q test (exact *p* value) followed by Marascuilo and McSweeney procedure (based on the minimum required difference) for multiple comparisons [17] was used to investigate if significant (*p* ≤ 0.05) purchase intent (PI) differences exist among the plate type and formulation combinations and compare the magnitude of the difference between the two formulations across plate types. Canonical discriminant analysis was used to determine the significance of the attributes’ liking on the discrimination among CFTC treatments. Linear regression and logistic regression models were used to predict OL and the odds of PI = Yes, respectively based on hedonic responses, plate type, and formulation. Cochran–Mantel–Haenszel (CMH) analysis was conducted for orange color, crunchiness, saltiness, and CF to test for the JAR scores’ homogeneous distribution across samples after controlling for differences among assessors followed by pairwise Stuart–Marxwell tests on significant (*p* ≤ 0.05) CMH tests. Subsequently, McNemar tests (with continuity correction factor) were conducted on significant pairwise Stuart–Marxwell tests collapsing the JAR categories (not enough vs. JAR + too much) to test for significant differences in the “not enough” category across treatments. Penalty tests and analyses [18] on the JAR ratings were performed to determine the effects of the sensory attributes on the liking of treatments. The total penalty score (TPS) for individual attributes was calculated by multiplying the percentage of “not-JAR” (either “not enough” or “too much”) by the corresponding mean drop (the difference between the mean liking score at “not-JAR” and the mean liking score at JAR [19]). Data analyses were performed using the XLSTAT (Addinsoft, New York, USA) statistical software version 2020 [20] and the Statistical Analysis Software (SAS) version 9.4 (Cary, NC, USA).

## 3. Results and Discussion

### 3.1. Physico-Chemical Properties of CFTC

Table 1 shows the instrumental fracturability (N), lightness (L*), redness (+a*), yellowness (+b*) values, the total color difference (ΔE), and sodium content for the CFTC formulations. Both formulations presented similar (*p* ≥ 0.05) fracturability, which is the maximum force to compress the product at the first significant peak in the texture analyzer probe’s first compression of the product, indicating no significant differences in instrumental crunchiness between formulations. On the other hand, CFTC formulations significantly (*p* < 0.05) differed in their lightness (formulation A= 61.57 vs. formulation B = 59.89) and yellowness values (formulation A = 46.68 vs. formulation B = 50.06), with formulation A color being brighter and less yellow than the color of formulation B. The obtained total color difference (ΔE = 4.81 > 2 threshold value) indicates noticeable color differences to the naked eye of untrained consumers [14,21] which may trigger other perceptual or hedonic differences between the formulations. Both formulations had similar sodium content (salt level) according to their nutritional label.

### 3.2. Sensory Evaluation of CFTC

#### 3.2.1. Consumers’ Acceptability and Purchase Intent (PI) of CFTC

Table 2 shows the sensory liking scores and PI results of the treatments. For all sensory attributes’ liking, formulation exerted a significant (*p* ≤ 0.05) effect, whereas the effect of plate type and the interaction between formulation and plate type were minimal (*p* ≥ 0.05). Previous studies have found that intrinsic product cues such as physical differences across products’ matrices (e.g., color differences) exert a stronger effect on consumer choices/preferences, which are useful predictors for actual purchase behavior in Western countries [22,23] than extrinsic product cues such as supplementary information, plate type, or other external determinants of product quality [24]. Other researchers have concluded that the relative importance of product extrinsic cues on consumers’ evaluations of product quality is highly dependent on product familiarity, enduring involvement, and price-reliant schema [25]. Depending on the degree of consumer-product interaction, different sensory characteristics become more important and elicit particular emotions. When consumers are well familiarized with the evaluated product, it is less likely that they will be affected by certain extrinsic cues like presentation format or serving displays [26]. Alternatively, Veale and Quester [1] concluded that intrinsic product attributes, even when experienced, may not be perceived, understand, or applied as intended when evaluated by consumers. Hence, differences in the surface roughness, transparency, weight, and other texture and visual aspects of the plates may not have been directly related to the perceived quality of the CFTC presentation format. Kpossa and Lick [27] found similar results when studying the effect of plate color on expectations and perceptions of pastries; plate color was not a significant factor influencing the actual perceptions (including hedonic and PI) of the products, only particular expectations.

Overall, the consumer’s liking scores were higher for formulation A within each plate type. Interestingly, formulation A treatments presented higher crunchiness liking scores than formulation B treatments (Table 2), although both formulations had similar instrumental fracturability (Table 1). This behavior could have reflected the occurrence of the “halo effect” because untrained panelists were recruited as is usually done for consumer studies. Panelists may have overestimated their crunchiness liking for formulation A treatments to justify their higher OL for this formulation [28]. Alternatively, this behavior could have been driven by the actual color differences between the formulations (A being brighter and less yellow than B), as it has been previously demonstrated that visual cues can alter textural perceptions and likings of food products [29]. Similarly, although sodium content for both formulations was similar, saltiness liking was higher for formulation A treatments. A similar trend was observed for OL and PI, possibly explained by the perceived differences in texture, saltiness, and OF between formulations although PI differences across formulations were significantly (*p* < 0.05) higher in plastic plates than in foam plates. Saltiness intensity expectations of formulation B treatments may have been negatively disconfirmed as participants were possibly expecting (stimulus and logical errors) a saltier taste from the more-yellowish formulation B treatments, which were, in fact, as salty as the formulation A treatments. Presumably, this disconfirmation may have led to decreased saltiness and OL scores which in turn affected the PI of formulation B treatments [30]. Similar results were reported in a study in which orange colorants (natural and artificial) were varied for mayonnaise-based dipping sauces in combination with a statement regarding the origin of the colorant [31]. Although dips contained the same sodium content, decreased liking scores were observed as colorant concentration was increased; such effect was attributed to the “horn effect”, a sensory bias that produces further penalization on a product’s attributes if its previously rated attributes were negatively perceived.

Table 3 shows the pooled within canonical structure from the canonical discriminant analysis of the hedonic ratings of all the evaluated sensory attributes and the treatments. This analysis provided the linear combinations (five canonical variates) of hedonic ratings with canonical coefficients that maximized (*p* < 0.0001) the distances among the treatments’ centroids. Liking of saltiness, crunchiness, OF, and OL (with canonical correlations, r, 0.58–0.94) discriminated the most among the treatments suggesting that these attributes are most critical for consumers’ overall sensory experience when consuming CFTC [32]. On the contrary, color and overall visual quality (which encompasses the serving inputs) contributed to a much lower extent in the discrimination across treatments, which is in line with the reported minimal effect of plate type factor on the liking of CFTC sensory attributes (Table 2).

Table 4 presents the regression coefficients and their probabilities from a fitted multiple linear regression model built to predict OL from overall visual quality, color, crunchiness, saltiness, and OF hedonic ratings and factors (plate type and formulation). The R-square of the fitted regression model was 0.78, which suggests additional inputs other than the sensory likings, formulation, and plate type evaluated in this study, may have contributed to the consumers’ OL ratings. Formulation, crunchiness liking, and saltiness liking were significant (*p* < 0.0001) regressors [33] for the OL prediction, but plate type was not. These results are congruent with the results from the liking scores (Table 2) and canonical discriminant analysis (Table 3).

When predicting PI (yes) of CFTC with a logistic regression model (Table 5) with sensory attributes ratings (excluding OL) and factors (plate type and formulation) as regressors, only the formulation, liking of crunchiness, saltiness, and OF significantly (*p* < 0.001) contributed to the PI prediction [34]. Plate type was not a significant predictor of PI, which agrees with the outcomes of the above-mentioned analyses (Table 2, Table 3 and Table 4). The odds of buying CFTC decreased by 64% when switching from formulation A to B (holding constant all other variables) whereas the odds of buying CFTC increased by 37, 49, and 60% when increasing one liking-rating unit in crunchiness, saltiness, and OF, respectively (holding constant all other variables).

#### 3.2.2. Just About Right (JAR) Responses and Total Penalty Scores (TPS) of CFTC

**Figure 2:**
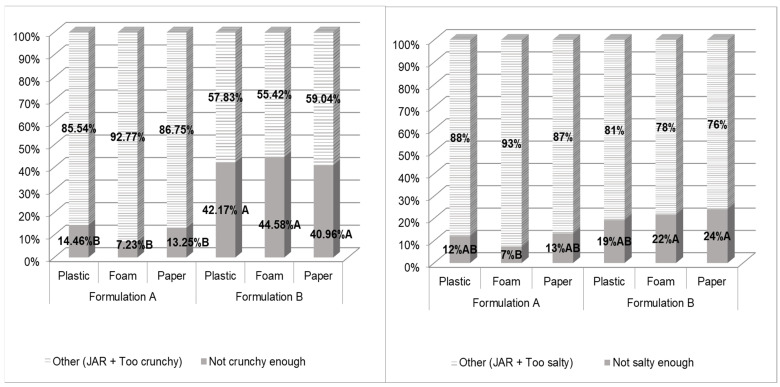
Just-About-Right (JAR) scores plot for treatments showing distributions of subjects’ responses for crunchiness (**left**) and saltiness (**right**). Pairwise comparison across samples’ not enough category was performed with McNemar test (applying continuity correction factor) only on samples with significantly different (*p* < 0.05 Cochran–Mantel–Haenszel and Stuart–Marxwell tests) JAR scores distribution. Treatments are described in Figure 1.

The JAR scores proportions (commonly expressed as percentages of panelists who selected each of the scale levels) evidence the perception of consumers’ attribute intensities (“not enough”, “JAR” or “too much”) relative to an internal ideal level/reference (“JAR”) [35]. Figure 2 depicts the frequency distribution of panelists’ ratings for the appropriateness of crunchiness and saltiness levels of the treatments over a JAR scale. The liking of these two attributes had a significant effect on discriminating among the treatments and on the prediction of overall liking (OL) and purchase intent (PI), albeit instrumentally similar across formulations. First, homogeneity of JAR scores distributions was tested across the treatments and rejected (*p* < 0.05) with a Cochran–Mantel–Haenszel (CMH) test. Subsequent treatment pairwise comparisons of JAR scores distribution were performed with Stuart Marxwell tests and were also significant (*p* < 0.05). Pairwise McNemar tests were then applied to compare the “not enough” categories across treatments by collapsing the other two categories (“JAR” and “too much”). From these tests, it was observed that formulation A was perceived as crunchier than formulation B. A brighter and lesser yellow color of formulation A may have been associated with crunchiness in the mindset of the participants in this study. Elicited previous experiences in which a crunchier perception was obtained for a similar product with that color characteristics was reported in previous studies [36,37].

On the other hand, a trend seems to indicate that formulation A was perceived as saltier than formulation B. However, the increased saltiness perception for formulation A was significant (*p* < 0.05) only for foam plates vs. formulation B presented in either foam or paper plates. Albeit plate type did not exert a significant effect on the liking, discrimination, OL, or PI prediction of CFTC, the visual color cue of CFTC possibly influenced their saltiness perception differently depending on the plate type.

Orange-color differences across formulations may have altered saltiness likings and intensity perceptions although actual OL differences across formulations may also have affected the liking ratings for other attributes with a similar level in both formulations (“halo effect”). Similar results were reported for expected and actual saltiness-intensity likings in a previous study with orange-colored dips [31].

**Figure 3:**
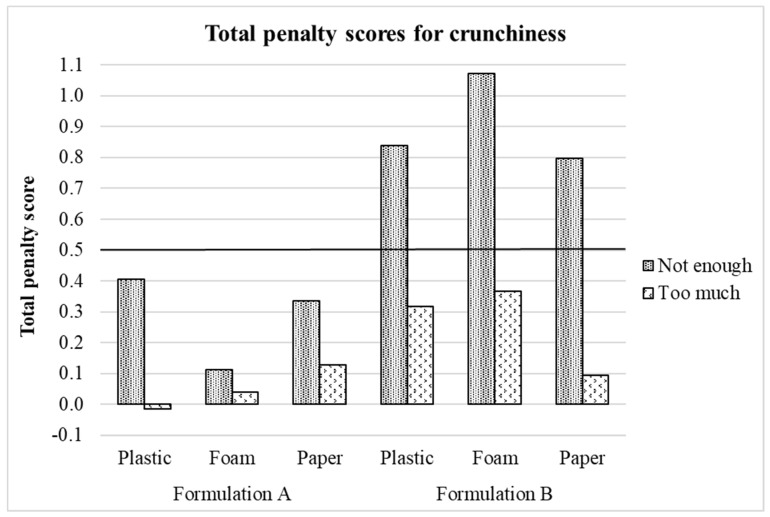
Crunchiness total penalty scores for treatments. Treatments are described in Figure 1.

**Figure 4:**
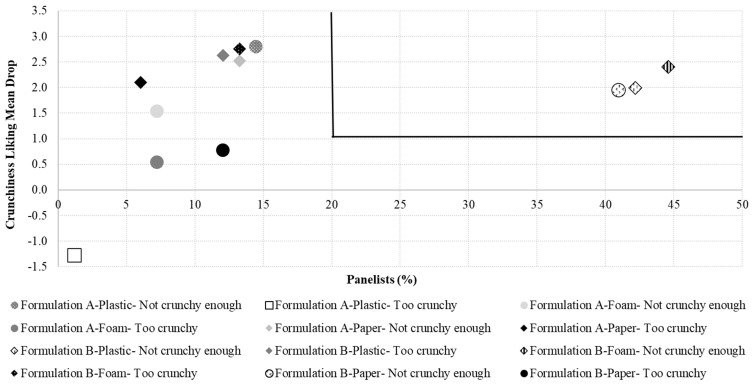
Penalty plot for treatments showing the mean drop in crunchiness liking due to “not crunchy enough” and “too crunchy” scores for crunchiness. Treatments are described in Figure 1.

Crunchiness TPS and mean drops originated from deviations of the panelists’ ideal-crunchiness-internal-reference level are illustrated in Figure 3 and Figure 4, respectively. In this case, both graphical tools show that the “not enough” crunchiness level of formulation B significantly penalized the crunchiness liking scores in all three plate types. When a TPS exceeds 0.5, the attribute should be reviewed to improve the product’s acceptability [38] while mean drops calculated in penalty analysis become concerning when they exceed 1–1.5 and represent at least 20% [39] of the panelists. These results agree with the ones derived from Figure 2 and in the previous sections of this study, crunchiness intensity was perceived differently across formulations (formulation B perceived as less crunchy) leading to the observed differences in crunchiness liking scores.

**Figure 5:**
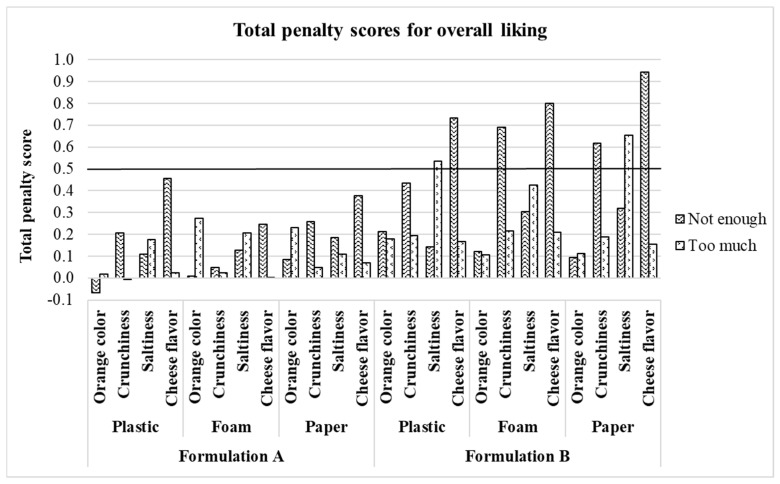
Treatments overall liking total penalty scores. Treatments are described in Figure 1.

**Figure 6:**
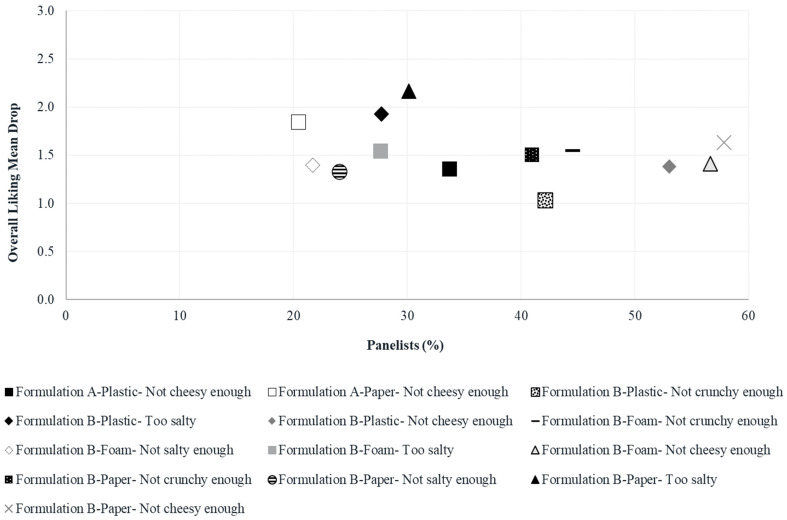
Penalty plot for treatments showing the significant mean drop in overall liking due to “not enough” and “too much” scores for sensory attributes. Treatments are described in Figure 1.

On the other hand, OL TPS (Figure 5) and mean drops (Figure 6) differed in the selection of the non-ideal categories of all the sensory attributes evaluated for the treatments that significantly penalized the OL scores. From Figure 5, it can be observed that all the significant TPS for OL originated from formulation B treatments: “too much salty” (plastic = 0.53 and paper = 0.65), “not enough cheese flavor” (plastic = 0.73, foam = 0.80 and paper = 0.94) and “not crunchy enough” (foam = 0.69 and paper = 0.62) whereas not significant TPS were obtained from formulation A treatments. In Figure 6, it is shown that most of the concerning mean drops (>1.5) for OL originated from formulation B treatments: “too much salty” (plastic = 27.7%, foam = 27.7% and paper = 30.1%), “not crunchy enough” (foam = 44.6% and paper = 41%) and “not enough cheese flavor” (paper = 57.8%). Instead, for formulation A, only “not enough cheese flavor” (paper = 20.5%) was a concerning level for OL although its frequency of selection was very close to the established threshold of interest (20%). These results evidence a negative implication on OL of formulation B treatments when perceived as “too much salty” or “not crunchy enough” although both formulations were instrumentally identical in both aspects.

## 4. Study Limitations

The sensory approach used in the present study involved the combined use of “Just-About-Right (JAR)” and hedonic scales to infer the level of product’s attributes that penalized the general and specific attributes product acceptability scores. This approach can provide meaningful insights for product optimization and further development and is still widely used among the food industry and academic researchers [18,40]. However, it has also been criticized by other researchers who demonstrated a significant effect of JAR questions on liking scores, thus discouraging the combined use in the same sensory session [28,41,42].

This study was conducted on two CFTC commercial samples that were formulated differently; hence, the potential effect of the visual color cue on crunchiness and saltiness perception and/or liking cannot be isolated from the possible occurrence of the “halo effect.” The halo effect is a common psychological error among untrained panelists (as is the usual case for consumer studies) in which other product attributes that were highly or poorly liked influence positively or negatively the attribute being evaluated, respectively.

Another limitation of this study was the number of subjects who participated in the consumer evaluation (N = 83), which is recommended to increase to at least N = 100 for future studies. Similarly, we recommended that, in future studies, the potential of extrinsic cues be evaluated in products with similar sensory properties or similar formulations and make sure the extrinsic cues being evaluated are in line with the product’s consumption context (i.e., considering differences among cultural practices and the scenario in which the experiment is being conducted).

## 5. Conclusions

Results through different statistical approaches were consistent in finding non-significant plate type effect and significant formulation effect on the sensory likings and PI of CFTC. Under the conditions of this study, the presentation of the CFTC in different serving displays seemed trivial in the consumer’s mind. In contrast, the intrinsic orange color cue of the CFTC potentially influenced crunchiness perception and possibly saltiness intensity perception, which mainly determined their acceptability and PI. Altering CFTC color towards brighter and lower yellow intensity can favor their crunchiness and saltiness perceptions towards ideal consumer levels, thereby positively influencing their liking and PI for fixed levels of salt content and other aspects of their formulation or processing. The findings from this study may be helpful to guide future product development towards healthier and more sustainable diets. Further research is recommended to understand specific mechanisms in which the orange color cue of CFTC affects crunchiness and saltiness intensity perception accounting for demographical variables and applying a wider range of color variation among the treatments.

## Figures and Tables

**Table 1:** Fracturability, color and sodium content of cheese-flavored tortilla chip formulations and standard error of the means (SEM) ^†^.

Attributes ^‡^	Formulation		SEM
	A	B	
Fracturability (N)	5.52 ^A^	6.14 ^A^	0.75
L*	61.57 ^A^	59.89 ^B^	0.01
a*	25.89 ^A^	24.07 ^A^	2.46
b*	46.68 ^B^	50.06 ^A^	0.08
ΔE	4.81		1.87
Sodium (Na mg/28 g) ^§^	210	220	-
Calories/28 g ^§^	150	140	-

^†^ Means data from six replicates samples (fracturability) and triplicate samples (L*, a*, b*). Different letters within a row represent significantly different samples (two-sample T-test *p* < 0.05). ^‡^ L* = (0 for darkness, 100 for lightness), a* = (− for greenness, + for redness), b* = (− for blueness, + for yellowness), ΔE = magnitude of total color difference between formulations. ^§^ According to nutritional label information.

**Table 2:** Sensory acceptability ^†^, standard error of the least-squares means (SEM), and purchase intent of treatments ^‡^.

Attributes ^§^	Plate Type and Formulation						SEM
	Plastic		Foam		Paper		
	Formulation A	Formulation B	Formulation A	Formulation B	Formulation A	Formulation B	
OVQL	7.16 ± 1.19 ^A^	6.35 ± 1.68 ^D^	6.94 ± 1.56 ^ABC^	6.41 ± 1.55 ^CD^	7.00 ± 1.40 ^AB^	6.57 ± 1.56 ^BCD^	0.16
OCL	6.80 ± 1.43 ^AB^	6.14 ± 1.68 ^C^	6.77 ± 1.59 ^AB^	6.33 ± 1.73 ^BC^	6.95 ± 1.46 ^A^	6.30 ± 1.73 ^BC^	0.18
CL	7.33 ± 1.42 ^A^	5.98 ± 1.75 ^B^	7.55 ± 1.06 ^A^	6.05 ± 1.86 ^B^	7.24 ± 1.46 ^A^	6.08 ± 1.73 ^B^	0.17
SL	6.96 ± 1.28 ^A^	5.80 ± 1.66 ^B^	6.98 ± 1.36 ^A^	5.86 ± 1.74 ^B^	6.71 ± 1.60 ^A^	5.84 ± 1.82 ^B^	0.17
OFL	6.94 ± 1.38 ^A^	5.51 ± 1.80 ^B^	7.16 ± 1.42 ^A^	5.65 ± 1.77 ^B^	7.13 ± 1.36 ^A^	5.43 ± 1.80 ^B^	0.18
OL	7.23 ± 1.12 ^A^	5.69 ± 1.61 ^B^	7.28 ± 1.13 ^A^	5.72 ± 1.58 ^B^	7.10 ± 1.27 ^A^	5.55 ± 1.81 ^B^	0.16
PI (%Yes) ^¶^	86.75 ^A^	37.35 ^B^	77.11 ^A^	44.58 ^B^	80.72 ^A^	39.76 ^B^	-
PI difference (%Yes) ^^^	49.40 ^A^		32.53 ^B^		40.96 ^AB^		-

^†^ Liking data are the least-squares means of N = 83 randomly selected consumers. Different uppercase letters within a row represent significantly (*p* < 0.05) different samples (Tukey’s means separation). ^‡^ Treatments are described in Figure 1. ^§^ OVQL = overall visual quality liking, OCL = orange color liking, CL = crunchiness liking, SL = saltiness liking, OFL = overall flavor liking, OL = overall liking, PI = purchase intent. ^¶^ Purchase intent data are the percentage of “Yes” category of N = 83 randomly selected consumers analyzed by two-sided Cochran’s Q test (exact *p* value) with Marascuilo and McSweeney procedure (multiple-pairwise-comparisons-minimum-required difference). ^^^ (%Yes Formulation A–%Yes Formulation B).

**Table 3:** Pooled within canonical structure (r) ^†^ explaining variables responsible for perceived differences between treatments ^‡^.

Attribute	Can 1	Can 2	Can 3	Can 4	Can 5
Overall Visual Quality Liking	0.3392	−0.2511	−0.3228	0.8131	0.1874
Orange Color Liking	0.3134	0.1535	−0.3148	0.7043	0.4553
Crunchiness Liking	0.7516	−0.0933	−0.5172	−0.2376	−0.0479
Saltiness Liking	0.5853	−0.2391	−0.1617	−0.0620	0.6808
Cheese flavor Liking	0.8511	0.2764	−0.1286	0.2346	0.0481
Overall Liking	0.9442	−0.0682	0.1454	0.0982	0.2487
Cumulative Variance	0.8895	0.9679	0.9868	0.9989	1.0000
Wilks’ Lambda *p* > F	<0.0001				

^†^ Canonical discriminant analysis of the hedonic ratings of all sensory attributes and treatments from N = 83 randomly selected consumers. ^‡^ Treatments are described in Figure 1.

**Table 4:** Multiple linear regression model ^†^ for overall liking (OL) prediction of cheese-flavored tortilla chips.

Parameter ^‡^	Estimate ^§^	*p* > ChSq ^§^	Type III LRT *p* > ChSq ^§^
Intercept	0.3626	0.0967	-
Paper plate	−0.1402	0.0901	0.2386
Foam plate	−0.0738	0.3732	
Plastic plate	-	-	
Formulation B	−0.2996	<0.0001	<0.0001
Formulation A	-	-	
OVQL	0.0468	0.3034	0.3037
OCL	0.0381	0.3774	0.3775
CL	0.2586	<0.0001	<0.0001
SL	0.2506	<0.0001	<0.0001
OFL	0.3798	<0.0001	<0.0001
Deviance *p*-value	1.00		

^†^ Based on maximum likelihood estimation, with overall model significance measured by likelihood ratio tests and individual parameters by Wald χ2 squared tests. Plastic plate and formulation A used as baseline categories. ^‡^ OVQL = overall visual quality liking, OCL = orange color liking, CL = crunchiness liking, SL = saltiness liking, OFL = overall flavor liking. ^§^ Coefficients and probabilities estimated using a model with all sensory-attribute likings and fixed effects (formulation and serving plate) as predictors and OL as the response variable.

**Table 5:** Logistic regression model ^†^ for purchase intent (PI) prediction of cheese-flavored tortilla chips.

Parameter ^‡^	Odds Ratio ^§^	*p* > ChSq ^§^	Type III LRT *p* > ChSq ^§^
Intercept	0.0010	<0.0001	-
Paper plate	0.8816	0.6831	0.6744
Foam plate	0.7615	0.3761	
Plastic plate	-	-	
Formulation B	0.3625	<0.0001	<0.0001
Formulation A	-	-	
OVQL	0.9831	0.9202	0.9202
OCL	1.1218	0.4757	0.4765
CL	1.3675	0.0005	0.0004
SL	1.4908	<0.0001	<0.0001
OFL	1.5984	<0.0001	<0.0001
Deviance *p*-value	0.8812		

^†^ Based on maximum likelihood estimation, with overall model significance measured by likelihood ratio tests and individual parameters by Wald χ2 squared tests. Plastic plate and formulation A used as baseline categories. ^‡^ OVQL = overall visual quality liking, OCL = orange color liking, CL = crunchiness liking, SL = saltiness liking, OFL = overall flavor liking. ^§^ Coefficients and probabilities estimated using a model with all sensory attribute likings (excluding overall liking) and fixed effects (formulation and serving plate) as predictors and PI as the response variable.

## Data Availability

The data that support the findings of this study are available from the corresponding author upon reasonable request.
